# Serotonin Transporter mRNA Expression Is Reduced in the Peripheral Blood Mononuclear Cells of Subjects with Major Depression but Normal in Fibromyalgia

**DOI:** 10.3390/brainsci13101485

**Published:** 2023-10-20

**Authors:** Gaël Villanueva-Charbonneau, Stéphane Potvin, Serge Marchand, Alexander McIntyre, Diane McIntosh, Alain Bissonnette, Alain Gendron, Charles-Édouard Giguère, Marie-Ève Koué, Édouard Kouassi

**Affiliations:** 1 Département de Pharmacologie et Physiologie, Université de Montréal, Montréal, QC H1T 1C8, Canada; 2 Centre de Recherche de l’Institut Universitaire en Santé Mentale de Montréal, Montréal, QC H1N 3V2, Canada; cedouard-giguere.iusmm@ssss.gouv.qc.ca (C.-É.G.); edouard.kouassi@umontreal.ca (É.K.); 3 Department of Psychiatry and Addiction, University of Montreal, Montreal, QC H3T 1J4, Canada; 4 Department of Surgery, Faculty of Medicine, University of Sherbrooke, Sherbrooke, QC J1K 2R1, Canada; serge.marchand@usherbrooke.ca; 5 Penticton Regional Hospital, Penticton, BC V0H 1K0, Canada; 6 Department of Psychiatry, University of British Columbia, Vancouver, BC V6T 1Z4, Canada; diane.mcintosh@telus.com; 7 Clinique du Campanile, Québec, QC G1X 4G6, Canada; abiss1@videotron.ca; 8 AstraZeneca Pharmaceuticals, Mississauga, ON L4Y 1M4, Canada; alain.gendron54@videotron.ca; 9 Department of Biochemistry and Molecular Medicine, University of Montreal, Montreal, QC H3T 1J4, Canada; mavek44@yahoo.fr; 10 Department of Medicine and Medical Specialities, University of Montreal, Montreal, QC H3T 1J4, Canada

**Keywords:** dopamine transporter, serotonin transporter, major depression, fibromyalgia, quetiapine

## Abstract

Background: Fibromyalgia (FM) and major depression disorder (MDD) frequently co-occur. Both disorders may share common serotonergic alterations, although there is less evidence of such alterations in FM. It is also unclear as to whether these alterations are persistent over time or transient. The objectives of this study were to (i) examine the changes in mRNA expression of serotonin transporter (SERT) on the surface of peripheral blood mononuclear cells (PBMCs) in FM, MDD, and the FM + MDD subjects compared to healthy controls, and to (ii) evaluate the effect of drug treatment on SERT expression. Methods: PBMCs were isolated from FM, MDD, FM + MDD, and control subjects. SERT expression was analyzed at the mRNA level via quantitative real-time polymerase chain reaction. Statistical analyses were performed using analyses of variance and linear mixed-effects models. Results: SERT mRNA expression was significantly reduced in MDD subjects compared to controls (*p* < 0.001), but not in FM nor in FM + MDD subjects. Although the drug treatments improved symptoms in FM, MDD, and FM + MDD subjects, they had no significant effect on SERT mRNA expression. Conclusions: These results corroborate the role of the SERT in the pathophysiology of MDD, but not in FM, and show that the decreased mRNA expression of SERT is a persistent, rather than transient, phenomenon.

## 1. Introduction

There has been an ongoing interest in identifying biomarkers related to mood and pain disorders. Despite high comorbidity rates, especially in the case of major depressive disorder (MDD) and functional pain syndromes, such as fibromyalgia (FM), few studies have focused on their frequent co-occurrence. Fibromyalgia (FM) affects between 0.2% and 6.6% of the population worldwide [1]. It is a complex disorder defined by widespread pain and tenderness in up to 19 areas of the body, lasting for at least 3 months [2]. According to the World Health Organization (WHO), MDD affects approximately 280 million people worldwide, and is one of the leading causes of disability [3]. It is characterised by the presence, for two or more weeks, of depressive mood and/or anhedonia and at least five of nine depressive symptoms. The diagnoses of MDD and FM commonly co-occur, with up to 80% of subjects suffering from FM meeting the diagnostic criteria for MDD [4], and 65% of MDD subjects reporting significant pain symptoms, including FM [5]. The high co-occurrence of FM and MDD suggests the need to identify biomarkers that are common and specific to both disorders.

The serotonin (5-HT) hypothesis, which states that the risk of developing depression is associated with a reduction in 5-HT levels, remains a prominent theory and a focus of research to develop more effective MDD treatments [6]. The main medication classes prescribed for the treatment of MDD, the selective serotonin reuptake inhibitors (SSRIs) and serotonin and norepinephrine reuptake inhibitors (SNRIs), directly bind to and modulate [7] the serotonin transporter (SERT). One aspect of the serotonin hypothesis proposes that SERT expression is elevated in MDD, leading to a reduction in 5-HT concentrations available in the synapse [8]. By blocking the SERT, SSRIs and SNRIs reduce 5-HT reuptake via synaptic cells, rapidly restoring the levels of 5-HT in the synapse and, over several weeks, various serotonin receptors respond to the heightened 5-TH level, leading to a range of downstream biochemical effects. Several studies have shown associations between functional polymorphisms of the promoter region of the SERT gene and the risk of developing depression [9].

While the serotonin hypothesis has been quite influential, unequivocally demonstrating its validity has posed a significant challenge. Several positron emission tomography (PET) studies have found that subjects with MDD have reduced SERT binding; although, a few of them did find an increase [10,11,12]. This lower SERT binding, compared to controls, has been interpreted as a compensatory response to decreased synaptic 5-HT levels associated with MDD. It must be noted, however, that the reduction in SERT binding was generally small and inconsistent across these studies, and it is unclear if these results are primary or secondary to antidepressant intake [13]. Likewise, genetic studies have failed to show an association between SERT genetic variants and MDD [14]. This lack of association has led some investigators to emphasize the importance of studying gene–environment interactions (e.g., epigenetics) [15]. Notably, the SERT is not only present in the brain but also in the peripheral blood, particularly on the surface of peripheral blood mononuclear cells (PBMCs), monocytes, and lymphocytes, where it has a broad role [16,17]. For example, various 5-HT receptors are expressed on the surface of T- and B-lymphocytes, and on antigen-presenting cells; their stimulation can contribute to inflammation, phagocytosis, migration, and cytokine production, as demonstrated in various human and animal models [18,19,20,21,22]. In recent years, a growing number of studies have examined the expression of the SERT on blood immune cells in MDD. Most studies detected a reduction in its expression in lymphocytes of MDD both at the mRNA [23] and protein levels [24,25,26,27], although increases in mRNA expression have also been observed in PBMCs [28]. The impact of drug treatment on these results remains to be determined, as well as the impact of important comorbid conditions, such as chronic pain.

Serotonin is also known to play a key role in the neurobiology of pain. It has been well established that 5-HT release from neurons in the rostro-ventral medulla dampen nociceptive afferents at the dorsal horn level of the spinal cord [29], producing diffuse analgesic effects. The involvement of 5-HT in pain modulation is of clear interest in the case of FM, considering that it is a chronic pain condition characterized by diffuse pain symptoms likely arising from deficient inhibitory conditioned pain modulation mechanisms [30]. So far, genetic studies that have examined the 5-HT_1A_ receptor and 5-HT_2A_ receptor gene polymorphisms, as well as studies measuring 5-HT blood levels, have produced mixed results [31,32,33]. In the case of the SERT, two genetic studies (one PET study and one study on gene expression) have also produced mixed results [34,35].

As compared to the treatment of MDD, SSRIs have shown only small benefits in FM [36], establishing the need to find other treatment alternatives. Several randomized-controlled trials have demonstrated that quetiapine, a second-generation antipsychotic, effectively treats mood symptoms in MDD [37]. Although this is less well established, there is growing evidence that quetiapine may be beneficial for the treatment of FM [30], as well as the treatment of subjects with co-morbid MDD and FM [38]. This drug increases 5-HT levels in the brain through its modulation of post-synaptic 5-HT_1A_ receptors and inhibition of 5-HT_2A_ receptors [39]. However, it is important to note that quetiapine has no direct effect on the SERT [40]. To date, there are no studies looking at the SERT mRNA expression in the population with a co-morbid of MDD and FM. Moreover, we are not aware of any available research regarding the effect of quetiapine on the mRNA expression of the SERT in the peripheral blood.

In view of the current state of knowledge, the main objectives of our work were as follows: (1) to measure the levels of mRNA expression in the PBMCs of the SERT in subjects affected by MDD or FM and those with both conditions, and (2) to evaluate the effect of quetiapine on the mRNA expression of the SERT in FM and FM + MDD, as well as the effect of various antidepressants in MDD. For exploratory purposes, we also measured the mRNA expression of the dopamine transporter (DAT) in all groups, as dopamine is a likely mediator of the cardinal symptoms of anhedonia in MDD. Additionally, dopamine may play a complex, and yet poorly understood, role in the pathophysiology of FM [41,42]. Based on the available literature, we expected to observe a reduction in the SERT mRNA expression levels in PBMCs in MDD, FM, and FM + MDD subjects.

## 2. Materials and Methods

### 2.1. Participants

Three study groups of patients were recruited, based on the three diagnostic categories: MDD with no chronic pain (n = 50), FM subjects (n = 55), and subjects with MDD and FM (n = 120). DSM-IV criteria were used for MDD selection and the American College of Rheumatology 1990 criteria for FM. Subjects in the FM + MDD group were washed out from their previous antidepressant treatment. In the FM group, subjects were allowed to continue their previous medication. In the MDD group, subjects were excluded if they met the criteria for any DSM-IV axis I disorder other than MDD. Across groups (FM, FM + MDD, and MDD), other exclusion criteria included: subjects currently prescribed an antipsychotic, pregnancy, female of childbearing potential without adequate contraception, current risk of suicide, neurologic disorders, substance use disorders, any unstable physical illness, and diabetes mellitus. A group of healthy subjects (n = 62) was also included, with no chronic pain and no history of a severe psychiatric disorder. In particular, the patient health questionnaire-9 (PHQ-9) was used to rule out any presence of depression. 

In the 3 subject groups, blood was taken on the first and last visit at the clinic and at different times according to studies (12 weeks for the FM study group, 8 weeks for the FM + MDD group, and 8 weeks for the MDD group). The subjects in the FM and FM + MDD arms were randomized in a double-blind, placebo-controlled fashion. In contrast, the MDD study was an open trial with no placebo. For the FM subjects, quetiapine was progressively introduced, as an add-on to previous analgesic medication, with a final flexible dose between 50 mg and 300 mg. For the FM + MDD subjects, a final dose of either 150 mg or 300 mg of quetiapine was gradually instituted. For the MDD group, treatments were heterogenous and prescribed at flexible doses, and included antidepressants like SSRIs (citalopram: daily dose range: 20–40 mg, and fluoxetine: daily dose range: 20–80 mg), venlafaxine (daily dose range: 75–225 mg), mirtazapine (daily dose range: 15–45 mg), or bupropion XL (daily dose range: 150–450 mg). For more information, please refer to published articles; for FM, Potvin et al. (2012) [30], and for FM + MDD, McIntyre (2014) [38].

### 2.2. Clinical Assessments

In all 3 study groups, depressive and anxiety symptoms were evaluated with the Hamilton depression rating scale (HAM-D) and the Hamilton anxiety rating scale (HAM-A), respectively [43,44]. The HAM-D and HAM-A were administered before and after pharmacological treatment in all 3 study groups (e.g., MDD, FM, and FM + MDD). FM symptoms were evaluated with the Fibromyalgia Impact Questionnaire (FIQ) [45]. The FIQ was administered before and after treatment in the FM + MDD and FM groups.

### 2.3. PBMC Isolation and qPCR

Blood samples were processed within 24 h of being collected in EDTA tubes. PBMCs were isolated via gradient centrifugation at 1850 g on Ficoll Paque (GE Healthcare, Mississauga, Canada). PBMC purity was determined with a cell counter (Coulter Ac T diff 2, Beckman Coulter, Montreal, Canada). Cells were stored at −80 °C in Trizol (Invitrogen, Burlington, Canada) until RNA extraction. Aliquots of one to two micrograms of the RNA samples were utilized for qPCR. After DNAse digestion (DNase I amplification grade, Invitrogen), inverse transcription was performed with an inverse transcriptase (iScript cDNA Synthesis Kit, Biorad, Saint-Laurent, Canada) for 5 min at 25 °C, 30 min at 42 °C, and 5 min at 85 °C. Complementary DNA (cDNA) was generated in the presence of different forward and reverse primers for the genes of interest: SERT and DAT, or primers for the housekeeping gene β2-microglobulin (β2). All primers were obtained from IDT, and their sequences are depicted in Table 1. The threshold cycle (CT) values obtained for the SERT or DAT were subtracted by the corresponding CT values of β2 to obtain the ΔCT values of the SERT or DAT. Relative SERT mRNA expression was calculated with the aid of the 2^−ΔΔCT^ method [46], using the clinical groups as the targets, and the healthy control group as a reference.

### 2.4. Statistical Analyses

All analyses were performed using R version 4.2.2 [47], using the package lmer [48] for mixed-effects analysis. The statistical threshold for significance was set at *p* < 0.05. First, we tested for a mean difference in the SERT mRNA expression level between the control group and the clinical groups (FM, MDD, and FM + MDD) using an analysis of variance. Given that there were significant differences, pairwise contrasts were performed between the groups using Tukey’s adjustment on the *p*-values. Second, for the FM and FM + MDD groups, we examined whether there was a mean change difference in the re-SERT for the subjects on the placebo compared to those receiving quetiapine using a linear mixed-effects model with a random effect on the intercept. For the MDD group (taking various antidepressants), we also used a linear mixed-effects model. Using the same model, scales measuring symptoms (pain, depression, anxiety, and global mental health) were compared from pre- to post-treatment to verify whether the symptoms improvements were greater in the quetiapine group as compared to the placebo group. Finally, in the case of the DAT mRNA expression level, it could not be detected in several participants. Hence, DAT mRNA expression was considered as a dichotomic variable, as it was either detected or not detected. In the case of the DAT mRNA expression level, logistic regression analyses were performed to examine potential between-group differences. Pairwise contrasts were performed between the groups using Tukey’s adjustment on the *p*-values.

## 3. Results

This section is divided by subheadings. It should provide a concise and precise description of the experimental results, their interpretation, as well as the experimental conclusions that can be drawn.

### 3.1. Clinical Findings

#### 3.1.1. Sociodemographic Differences

At baseline, there were significant differences in age between groups (FM, N = 55: 49.6 years ± 10.3; FM + MDD, N = 120: 50.7 years ± 9.5; MDD, N = 50: 44.6 years ± 12.2; and controls, N = 62: 38.0 years ±12.8; *p* < 0.001). The sex ratio was also significantly different between groups (FM: 100% of females; FM + MDD: 97.2% of females; MDD: 48.8% of females; and controls: 25.8% of females; *p* < 0.001).

#### 3.1.2. Pre- and Post-Treatment Effects

In the FM group, 25 subjects received quetiapine, while 61 subjects received quetiapine in the FM + MDD group. As illustrated in Supplementary Table S1, we observed a reduction in FM-related and depressive symptoms after treatment with quetiapine, relative to placebo, across the FM and FM + MDD groups (FIQ: *p* = 0.002; HAM-D: *p* = 0.003). There was a similar trend in the case of anxiety symptoms, which failed to achieve significance (*p* = 0.08). Regardless of drug status (quetiapine vs. placebo), there was a significant effect of time on FM, depressive, and anxiety symptoms across the FM and FM + MDD groups (all *p* < 0.001). For more information, please refer to Potvin et al. (2012) [30] and McIntyre et al. (2014) [38]. As illustrated in Supplementary Table S1 and Supplementary Figure S1, for MDD subjects, we observed significant improvements in depressive and anxiety symptoms after treatment (HAM-D: *p* < 0.001; HAM-A: *p* < 0.001).

### 3.2. Differences in SERT mRNA Expression between Groups before Treatment

**Figure 1:**
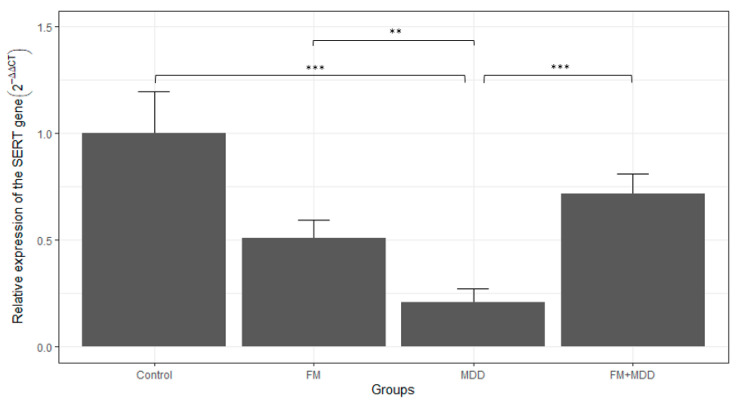
Relative expression of the SERT mRNA level by groups at baseline, using the 2^−ΔΔCT^ method and the control group as a reference. Error bars represent one standard error. ** *p* < 0.01, and *** *p* < 0.001.

We observed a minor reduction in SERT mRNA expression in FM subjects relative to healthy subjects, but this potential difference failed to reach statistical significance (*p* = 0.061) (Figure 1). There was a significant reduction in the SERT mRNA expression level in the MDD group compared to the control group (*p* < 0.001) (Figure 1). This reduction had a magnitude of −2.249 ± 0.415 amplification cycles via PCR. Assuming a doubling of the amplicon per amplification cycle, this result indicates that the SERT gene expression level was about five times less expressed in the MDD subjects compared to healthy controls. We also observed a significant decrease in the SERT gene expression level in the MDD group when contrasted to the two other subject groups: FM by a factor of −1.279 ± 0.380 cycles (*p* < 0.01), and −1.768 ± 0.342 for FM + MDD (n = 117) (*p* < 0.001), respectively. This indicates that the SERT was two and three times less expressed in MDD than in FM and FM + MDD, respectively. Finally, there was no difference noted between the FM + MDD group and the control group (*p* = 0.514) (Figure 1). Of note, these results remained significant after controlling for age differences (MDD vs controls: *p* < 0.001; MDD vs. FM: *p* < 0.01; MDD vs. FM + MDD: *p* < 0.001) using an analysis of covariance.

Considering that there were significant differences in the sex ratio between these groups, we performed secondary analyses restricted to only female participants. As in the primary analysis, differences were found between the MDD subjects and the controls (*p* = 0.0001), as well as between the MDD and FM subjects (*p* = 0.002) (Supplementary Figure S2).

### 3.3. Changes in the SERT mRNA Expression Level after Treatment

**Figure 2:**
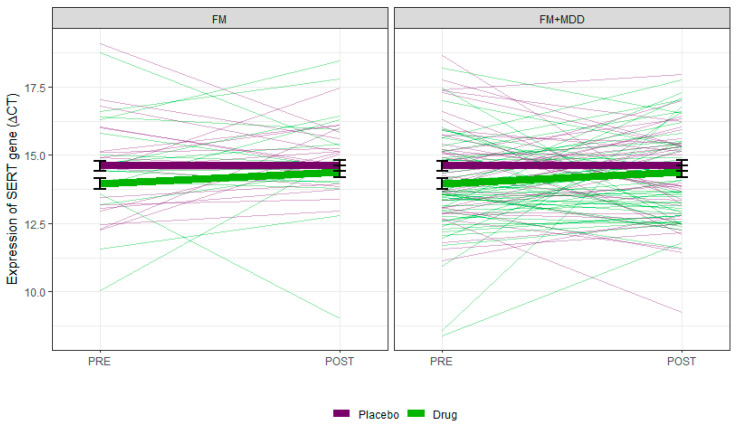
Changes in the expression of SERT mRNA from pre- to post-treatment. Thin lines in the background represent individual changes, and the solid thick lines represent the means from a linear mixed-effects model. Error bars represent one standard error (SE).

In the FM group, SERT mRNA expression levels were analyzed in 20 subjects receiving placebo versus 18 receiving quetiapine after 12 weeks of treatment. In the FM + MDD group, SERT mRNA expression levels were analyzed in 43 subjects taking placebo and 51 taking quetiapine after 8 weeks of treatment. Across both groups, no significant difference was observed in the SERT mRNA expression level between subjects taking the placebo and those receiving quetiapine after treatment (*p* > 0.05) (Figure 2). Regardless of drug status (quetiapine vs. placebo), there was a no effect of time on the SERT mRNA expression level across the FM and FM + MDD groups (*p* > 0.05) Due to loss to follow-up and missing blood samples, the SERT mRNA expression level was only analyzed in 16 MDD subjects after 8 weeks of treatment. The comparison of SERT mRNA expression of MDD subjects before and after treatment showed no significant difference after treatment (*p* > 0.05).

### 3.4. Differences in the DAT mRNA Expression Level

**Figure 3:**
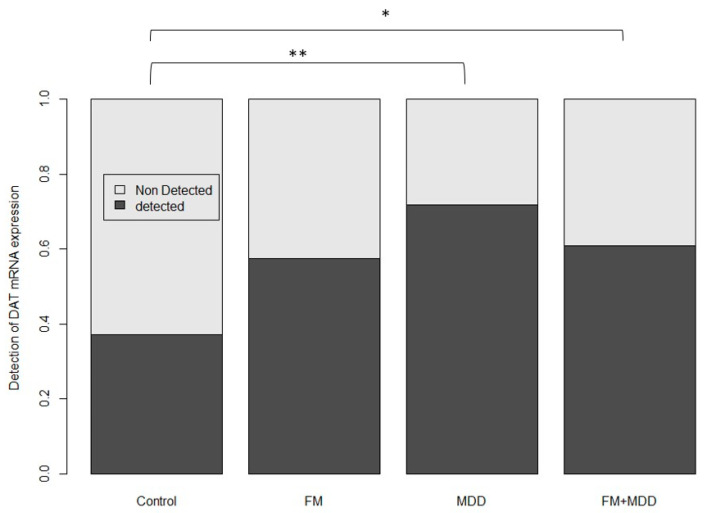
Boxplot of the expression of the DAT mRNA by groups at baseline using a categorical approach, with a cut-off of >50. The more frequent the mRNA was detected, the more it was considered expressed, and vice-versa. * *p* < 0.05, ** *p* < 0.01.

We used a categorical analysis to evaluate DAT mRNA expression. At baseline, DAT mRNA expression was detectable in 37.3% of controls, 55.6% of FM subjects, 62.5% of FM/MDD subjects, and 69.0% of MDD subjects. DAT mRNA expression was more frequently detected in the MDD subjects relative to controls (*p* = 0.002), and more frequently detected in the FM + MDD subjects relative to controls (*p* = 0.01) (Figure 3). We observed that DAT mRNA expression was detected in a higher percentage of FM subjects as compared to the controls; however, this result was non-significant (*p* = 0.18) (Figure 3). Similar group differences were observed in DAT mRNA expression levels after drug treatment.

Considering that there were significant differences in the sex ratio between groups, we performed secondary analyses restricted to only female participants. As in the primary analysis, differences were found between the MDD subjects and controls (*p* = 0.01) (Supplementary Figure S3).

## 4. Discussion

The main aim of the present study was to measure serotonin transporter (SERT) and dopamine transporter (DAT) mRNA expression levels in PBMCs in MDD, FM, and subjects with both conditions. As a secondary objective, we sought to determine the impact of different antidepressants (mainly quetiapine) on SERT and DAT mRNA expression levels. Our results showed a decrease in SERT mRNA expression in the MDD subjects, but not in the FM subjects. No change over time was detected after treatment, even though significant clinical improvements were observed in all three study groups for both depressive and/or FM symptoms. In comparison, the expression of DAT mRNA was difficult to detect in several participants. Nevertheless, we were able to observe an increase in DAT mRNA expression in the MDD subjects, with smaller effects being observed in the FM + MDD subjects.

The main finding of the present study was the observation of a decrease in SERT mRNA expression in the MDD subjects. This result is consistent with the most accepted serotonergic model of depression and with the findings of previous studies in the field. Indeed, in most cases, these studies have shown that there is a decrease, rather than an increase, in SERT expression in MDD using blood lymphocytes as a cell source, both at the mRNA [23] and protein levels [24,25,26,27,49]. This may seem counterintuitive, given that a decrease in SERT mRNA expression would normally result in an increased availability of 5-HT in the peripheral blood. However, in previous studies with similar results, the authors interpreted this finding by considering the alterations in SERT mRNA expression as not primary, but rather as secondary, to the abnormal amounts of 5-HT available in the peripheral blood in MDD subjects [25]. In this light, the decrease in SERT mRNA expression would represent a neuroimmune adaptive response to the presence of a reduced amount of 5-HT in the peripheral blood in MDD [50]. While previous studies in this field have been cross-sectional [23,24,25,26,27,49], the present study stands out for its longitudinal design. Our results showed no change in SERT mRNA expression in any of the three study groups, including the MDD group, where the SERT mRNA expression was abnormal at baseline. In contrast with the other two groups, which were treated with a medication which had no affinity for SERT (e.g., quetiapine) [51], the MDD group was primarily treated with antidepressants (e.g., SSRIs and venlafaxine) known to inhibit the SERT. The fact that no normalization of SERT mRNA expression was observed in the MDD subjects over time suggests that the reduction in SERT mRNA expression represents a stable, rather than a transient, effect. This interpretation is reinforced by the fact that no change in SERT mRNA expression was observed in the MDD group before or after treatment, even though the anxiety and depressive symptoms of these subjects improved over time.

Regarding the lack of association between SERT mRNA expression and FM, this result must be considered in light of previous studies evaluating the role of 5-HT in FM, which have produced conflicting results [52]. For instance, our research team previously studied blood 5-HT levels in FM and the serotonin transporter promoter region (5-HTTLPR) polymorphism and found no associations with FM in both cases [30,53,54]. Serotonin does not exhibit simple effects on pain. Indeed, the preclinical literature has shown that brainstem 5-HT is involved in both pain inhibition and pain facilitation [55], as 5-HT produces different effects on pain depending on the receptors to which it binds. While 5-HT_1A_ receptor agonists produce pain relief, 5-HT_2A_ and 5-H_T3_ receptor agonists promote pain [56]. The SERT regulates the amount of 5-HT available in the synaptic cleft, but as the 5-HT receptors have opposing effects on pain, the measurement of SERT mRNA expression may lack the specificity required to demonstrate the involvement of 5-HT in the pathophysiology of chronic pain conditions, such as FM.

Since the expression of the DAT mRNA was undetectable in many participants, even after 50 cycles of amplification in the qPCR reactions, the results regarding DAT mRNA expression must be cautiously interpreted. Nevertheless, our results indirectly suggest that DAT mRNA expression levels are increased in MDD. One of the cardinal symptoms of MDD is anhedonia. In the past, it has been proposed that anhedonia might result from reduced dopamine release in the brain reward system [57]. In support of this model, several animal studies have revealed that the administration of dopamine D_2_ receptor antagonists in the striatum attenuates the reinforcing effects of various psychoactive substances [58]. In humans, several functional neuroimaging studies have shown that striatal activity is reduced in MDD subjects when they anticipate or receive a reward [59]. 

Despite a few negative findings, genome wide association studies and meta-analyses have shown an association between MDD and certain dopamine-related gene variants [60,61]. Although these results are heterogeneous, several PET studies have shown alterations in striatal DAT availability in MDD [62]. Finally, randomized controlled trials have shown that bupropion (a weak DAT inhibitor) and several D2 receptor partial agonists (e.g., aripiprazole) are effective in treating MDD [63]. In theory, the increased DAT mRNA expression level could result in reduced amounts of dopamine in the synaptic cleft, including in the brain reward system, which could explain the symptoms of anhedonia experienced by subjects with MDD. However, in the current study, DAT mRNA expression was measured in PBMCs; thus, our results may not necessarily reflect changes occurring in the brain. As for the increased DAT mRNA expression levels found in the FM + MDD subjects, this result was barely significant, suggesting that the observed effect is rather small. It must be considered that the role of dopamine in pain is complex and remains to be clarified [64], and that the dopaminergic alterations that have been described in FM remain preliminary and need to be confirmed [65].

The current study has several strengths, namely the inclusion of a relatively large sample of subjects (total N = 225) with MDD, FM and FM + MDD. Subjects in all three groups were assessed before and after antidepressant treatment. Finally, two out of the three groups were assessed in a randomized, placebo-controlled manner. Despite these strengths, the study has some limitations that must be acknowledged. First, there were significant differences in the age and sex ratio between groups. Considering that age and sex may influence the serotoninergic system [66,67], the between-group difference in socio-demographic variables may explain the reduced SERT mRNA expression levels that were observed in the MDD subjects relative to controls. However, this possibility seems unlikely, as we performed sub-analyses controlling for the effects of age and sex, which showed that the decrease in SERT mRNA expression remained significant in MDD despite the addition of these covariates. Another limitation of the current study is related to the fact that the FM and FM + MDD groups were not treated with the same antidepressant (e.g., quetiapine) as the MDD group, which received an assortment of antidepressants (including the SSRIs). Although quetiapine exhibits affinities for several 5-HT receptors [39], the fact that this drug has no known affinity for the SERT may explain the lack of change in the SERT mRNA expression level during treatment in the FM and FM + MDD groups. However, the MDD subjects were treated with antidepressants having affinities for SERT, and no change in SERT mRNA expression was detected in this group. Finally, it must be acknowledged that SERT mRNA was only measured in 16 MDD subjects after the 8-week treatment. Thus, we may have statistical power to detect changes in SERT mRNA expression before and after antidepressant treatment.

## 5. Conclusions

Consistent with the serotonergic model of depression, the results of the present study showed a decrease in SERT mRNA expression and no effect of quetiapine on this outcome, suggesting that the increase in SERT mRNA expression represents a trait, rather than a state effect. On the other hand, we did not observe any alteration of SERT mRNA expression in FM. In a preliminary manner, we also observed an increase in DAT mRNA expression in MDD (and possibly in FM); however, this result was not robust, due to the difficulty in detecting the DAT in many participants. Future investigations are required to measure the mRNA expression levels of several 5-HT and dopamine receptors, before and after the administration of different types of antidepressants, while paying attention to the potential confounding effects of age and sex differences.

## Figures and Tables

**Table 1:** Oligonucleotides of primers of the SERT, DAT, and β2-microglobulin.

Species	Sense	Antisense	Amplicon
SERT (NM_001045.6)	GTGGCCAAAGACGCAGGTC (1494–1512)	CTCATCCAGCACAGCCGTGATC (1664–1643)	171 bp
DAT (NM_001044.5)	CTGCGAGGCGTCTGTTTGGATTG (1053–1075)	GTGGTGACAATCGCGTCCCTGTAG (1187–1164)	135 bp
β2-microglobulin (NM_004048.4)	CACGTCATCCAGCAGAGAATGG (122–143)	GATGCTGCTTACATGTCTCGATCC (398–375)	277 bp

## Data Availability

The data that support the findings of this study are available upon reasonable request to the corresponding author, S.P., but are only redistributable to researchers engaged in IRB-approved research collaborations.

## Supplementary Materials

The following supporting information can be downloaded at: https://www.mdpi.com/article/10.3390/brainsci13101485/s1. Table S1. Mean (sd) of the symptom scale pre and post by group for the placebo and drug group. The measured symptoms were taken from Fibromyalgia Impact Questionnaire Total (FIQ-Total), the Hamilton Depression (HAM-D), and Anxiety (HAM-A). Supplementary Figure S1. Changes in clinical symptoms from pre- to post-treatment for the MDD group. Thin lines in the background represent individual changes and the solid thick lines represent the means from a linear mixed-effect model. Error bars represent one standard error (SE). The measured symptoms were taken from the Hamilton Depression (HAM-D) and Anxiety (HAM-A). Supplementary Figure S2. Relative expression of the SERT mRNA by groups at baseline, using the 2^−ΔΔCT^ method and the control group as reference. This analysis was restricted only to female participants across groups. Error bars represent one standard error. *** *p* < 0.001. Supplementary Figure S3. Boxplot of the expression of the DAT mRNA by groups at baseline, using a categorical approach. This analysis restricted only to female participants across groups. The Y axis represents the level of the DAT gene detection. The more frequently the mRNA is detected, the more it is considered expressed, and vice-versa. * *p* < 0.05.
