# Supplementary material for: Serotonin Transporter mRNA Expression Is Reduced in the Peripheral Blood Mononuclear Cells of Subjects with Major Depression but Normal in Fibromyalgia
Source: Brain Sci. 2023 Oct 20;13(10):1485. doi: 10.3390/brainsci13101485 (PMC10605238; doi:10.3390/brainsci13101485)
Supplement: Supplementary file 1 [file brainsci-13-01485-s001.zip › brainsci-2654723-supplementary.pdf]

Supplementary Material

Supplementary Table S1: Mean (sd) of the symptom scale pre and post by group for the placebo and drug group. The measured symptoms were taken from Fibromyalgia Impact Questionnaire Total (FIQ-Total), the Hamilton Depression (HAM-D), and Anxiety (HAM-A).

		Placebo		Quetiapine	
		Pre	Post	Pre	Post
FIQ-Total	FM	63.8 (13.5)	61.3 (15.3)	59.7 (12.4)	54.5 (16.6)
	FM+MDD	75.6 (13.7)	65.2 (16.5)	77.2 (14.0)	60.8 (22.0)
HAM-D	FM	12.6 (3.8)	12.2 (3.6)	11.1 (4.6)	9.1 (5.5)
	MDD	-	-	26.2 (7.5)	13.6 (8.1)
	FM+MDD	24.6 (1.5)	15.9 (4.8)	25.1 (2.3)	12.3 (5.6)
HAM-A	FM	11.5 (3.9)	10.3 (3.4)	10.3 (4.7)	8.8 (4.4)
	MDD	-	-	25.7 (7.6)	12.2 (8.2)
	FM+MDD	23.5 (4.6)	14.7 (5.7)	24.0 (4.5)	11.9 (6.4)

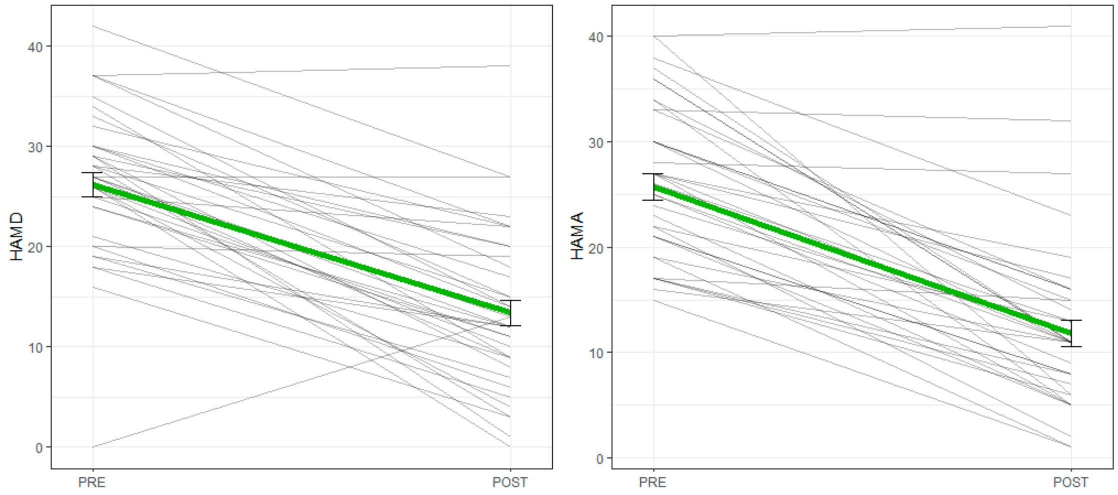

Supplementary Figure S1: Changes in clinical symptoms from pre- to post-treatment for the MDD group. Thin lines in the background represent individual changes and the solid thick lines represent the means from a linear mixed-effect model. Error bars represent one standard error (SE). The measured symptoms were taken from the Hamilton Depression (HAM-D) and Anxiety (HAM-A).

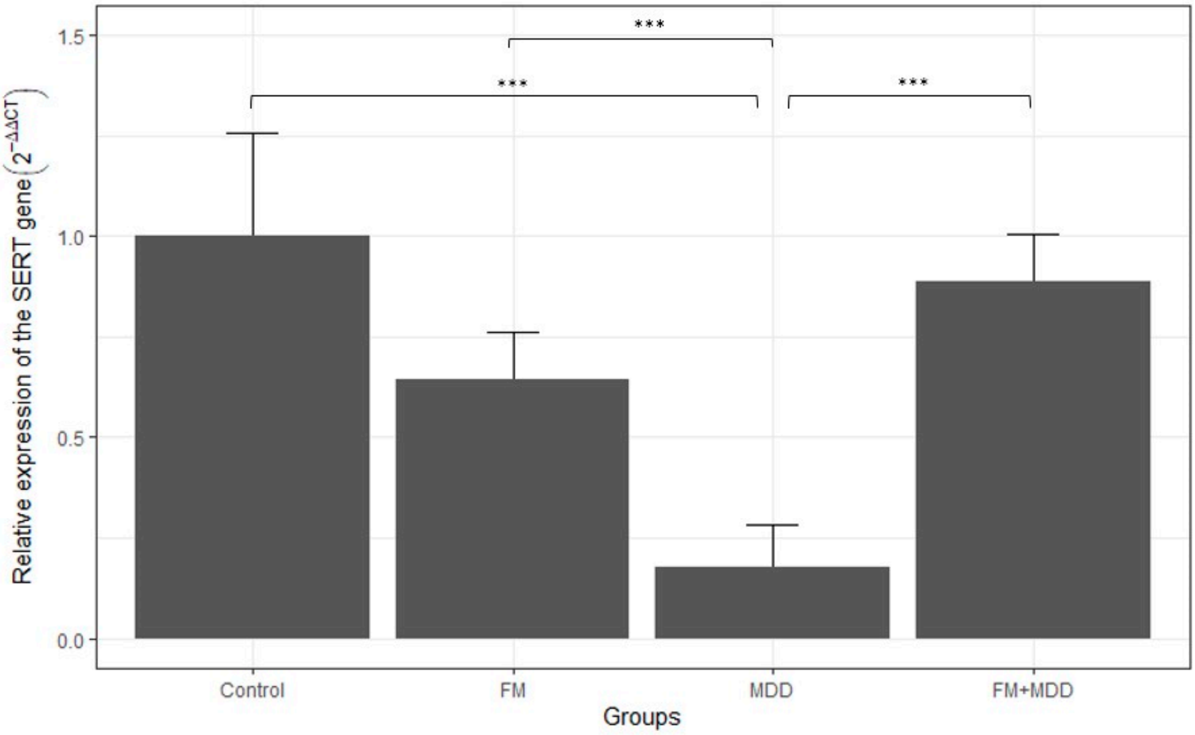

Supplementary Figure S2: Relative expression of the SERT mRNA by groups at baseline, using the $2^{-\Delta\Delta CT}$ method and the control group as reference. This analysis was restricted only to female participants across groups. Error bars represent one standard error. *** p<0.001.

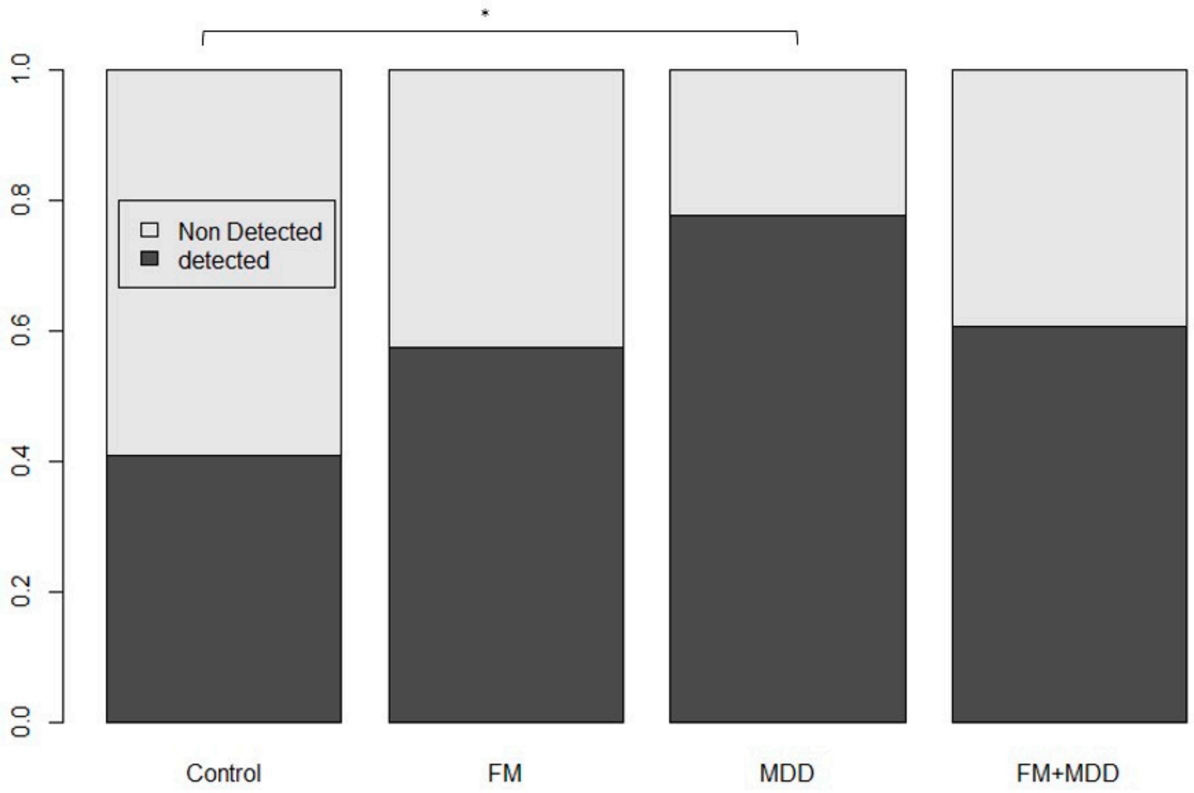

Supplementary Figure S3: Boxplot of the expression of the DAT mRNA by groups at baseline, using a categorical approach. This analysis restricted only to female participants across groups. The Y axis represents the level of the DAT gene detection. The more frequently the mRNA is detected, the more it is considered expressed, and vice-versa. * $p<0.05$.
